# Therapeutic efficacy of leuprorelin acetate combined with rhGH in girls with ICPP and Its impact on body height and secondary sexual characteristics

**DOI:** 10.12669/pjms.42.6.14211

**Published:** 2026-06

**Authors:** Jinxia Xu, Qinghuang Zeng, Yuanbin Xu

**Affiliations:** 1 Jinxia Xu, Department of Paediatrics, Affiliated Hospital of Putian University, Putian 351100, Fujian, China; 2 Qinghuang Zeng, Department of Paediatrics, Affiliated Hospital of Putian University, Putian 351100, Fujian, China; 3 Yuanbin Xu, Department of Paediatrics, Affiliated Hospital of Putian University, Putian 351100, Fujian, China

**Keywords:** Body Height, Idiopathic Central Precocious Puberty, Leuprorelin Acetate, Recombinant Human Growth Hormone, Secondary Sexual Characteristics, Therapeutic Efficacy

## Abstract

**Objective:**

To investigate the therapeutic efficacy of leuprorelin acetate combined with recombinant human growth hormone(rhGH) in girls with idiopathic central precocious puberty(ICPP) and its impacts on body height and secondary sexual characteristics.

**Methodology:**

This was a retrospective study. One hundred and fifty-four girls with ICPP admitted to Affiliated Hospital of Putian University between November 2022 to November 2025 were randomly allocated into two groups: the control group (leuprorelin acetate monotherapy) and the study group (leuprorelin acetate plus rhGH), with 77 cases in each group. Therapeutic efficacy, growth parameters, secondary sexual characteristics, serum sex hormone levels, and adverse reactions were compared between the two groups.

**Results:**

The overall response rate in the study group was markedly higher than that in the control group(*P*<0.05). Following treatment, body height and weight of girls in the study group were significantly greater than those in the control group, while the bone age/chronological age (BA/CA) ratio, uterine volume, ovarian volume, breast size, and serum levels of estradiol(E2), follicle-stimulating hormone (FSH), and luteinizing hormone (LH) were notably lower in the study group (all *P*<0.05).

**Conclusion:**

Combination therapy with leuprorelin acetate and rhGH yields substantial therapeutic benefits in girls with ICPP. It may effectively regulate sex hormone levels, facilitates improvements in growth and secondary sexual characteristics, and demonstrate favorable clinical safety profiles.

## INTRODUCTION

Idiopathic central precocious puberty (ICPP) represents a prevalent developmental disorder in pediatric endocrinology, characterized by the premature activation of the hypothalamic-pituitary-gonadal axis (HPGA) and classified as gonadotropin-releasing hormone (GnRH)-dependent precocious puberty. Clinically, it manifests as the development of secondary sexual characteristics before 7.5 years of age in girls and nine years of age in boys, in the absence of identifiable organic causes.^1^ By prematurely activating the HPGA, ICPP induces early secretion of sex hormones, accelerated sexual organ development, and significant bone age advancement, ultimately compromising adult height and potentially contributing to psychological and behavioral issues.^2^ Epidemiological surveys indicate that 80%-90% of pediatric ICPP cases in China are of idiopathic type, requiring comprehensive diagnosis based on bone age assessment, sex hormone detection, and imaging examinations.^3^

The current standard treatment is centered on gonadotropin-releasing hormone analogs (GnRHa) such as leuprorelin acetate, which sustainably inhibit pituitary gonadotropin secretion, reduce sex hormones to prepubertal levels, and delay skeletal maturation.^4^ However, while GnRHa monotherapy effectively controls sexual development, its efficacy in improving adult height is limited-approximately 20%-30% of children fail to reach their genetic target height due to insufficient growth hormone secretion or premature epiphyseal closure.^5^ Additionally, some children may develop treatment resistance or disease recurrence, underscoring the critical need for refined therapeutic approaches. Recombinant human growth hormone (rhGH) has a well-established role in promoting longitudinal bone growth and improving muscle mass.^6^

Recent studies have shown that combining rhGH with GnRHa achieves synergistic effects through a dual mechanism: rhGH directly stimulates the proliferation of epiphyseal chondrocytes and extends the growth window.^7^ Therefore, this study focuses on the combined application of leuprorelin acetate and rhGH, aiming to systematically evaluate its therapeutic efficacy and impacts on height growth and secondary sexual characteristics in girls with ICPP.

## METHODOLOGY

This was a retrospective study. One hundred fifty-four girls with ICPP who were admitted to Affiliated Hospital of Putian University between November 2022 to November 2025 were randomly divided into the control group and the study group, with 77 cases in each group. All participant data included the medical data from our hospital information and management system, collected their various information of them.

### Ethical approval:

The study was approved by the Institutional Ethics Committee of Affiliated Hospital of Putian University (No.:2025302; Date: November 05, 2025), and written informed consent was obtained from all participants’ guardians.

### Inclusion criteria:

- Children who met the diagnostic criteria for ICPP.^8^
- Female children aged 6-11 years.
- Those who were newly diagnosed and those who had not received any prior treatment for precocious puberty(e.g., GnRHa, growth hormone, traditional Chinese medicine, or hormonal agents).
- Whose those guardians had signed the informed consent.

### Exclusion criteria:

- Children who had severe organic diseases (e.g., abnormalities in cardiac, pulmonary, hepatic, or renal function).
- Those who had chromosomal abnormalities or genetic metabolic disorders (e.g., Turner syndrome, congenital adrenal hyperplasia).
- Those who had organic precocious puberty induced by central nervous system lesions (including hypothalamic/pituitary tumors, craniocerebral trauma, inflammation), adrenal diseases, gonadal tumors, or other etiologies.
- Those who had peripheral precocious puberty (e.g., exogenous sex hormone exposure, ovarian cysts, adrenal cortical hyperplasia).
- Those who were concurrently using drugs affecting growth and development (e.g., glucocorticoids, immunosuppressants, antiepileptic drugs).
- Those who had recently used (within three months) sex hormone-containing drugs or health products (e.g., contraceptives, royal jelly, breast enhancement creams).

### Therapeutic regimens:

Monotherapy with leuprorelin acetate was administered to the control group. The initial dose was given as a subcutaneous injection once every four weeks, with dosage titration based on body weight: 60-100 μg/kg for children with body weight ≤30 kg; a fixed dose of 3.75 mg per injection for those with body weight >30 kg. For children with rapid sexual development progression or menarche, an additional booster dose was administered 14 days after the initial injection. Subsequent doses could be adjusted to 90 μg/kg or maintained at 3.75 mg per injection, given once every 28 days, with a total treatment course of two years.

The study group received combined therapy with leuprorelin acetate and rhGH. Subcutaneous rhGH was administered once daily, with a total weekly dose of 0.3-0.4 mg/kg divided into seven daily injections (i.e., approximately 0.04-0.05 mg/kg per day). Injections were given 0.5 hours before bedtime, and the treatment course was consistent with that of the control group.

### Outcome measures:

- ***Therapeutic efficacy evaluation:*** Markedly effective: Breast development ceased or regressed to Tanner Stage-I (prepubertal state), with cessation or regression of pubic/axillary hair growth; GnRH stimulation testing revealed an luteinizing hormone (LH) peak of <5.0 U/L, pelvic ultrasound showed a uterine volume ≤2 mL, and predicted adult height (PAH) increased by >5 cm. Effective: Slow progression of breast development (no progression or only 1-stage advancement in Tanner staging) and reduced growth rate of pubic/axillary hair; GnRH stimulation testing revealed an LH peak of 5.0-7.0 U/L, partial reduction of sex hormone levels (still higher than normal prepubertal levels), ovarian volume <3 mL, and PAH increased by 3-5 cm. Ineffective: Failure to meet the above “effective” criteria.
- ***Growth parameters:*** Bone age (BA) was evaluated via X-ray radiography. Body height and weight of children in both groups were measured, and the bone age/chronological age (BA/CA) ratio was calculated.
- ***Secondary sexual characteristics:*** Uterine volume and ovarian volume were measured by pelvic ultrasonography, and breast size was assessed via X-ray imaging.
- ***Serum sex hormone levels:*** Estradiol (E2) level was quantified using enzyme-linked immunosorbent assay (ELISA), while follicle-stimulating hormone (FSH) and LH levels were measured by chemiluminescent immunoassay (CLIA).
- ***Adverse reactions:*** The occurrence of adverse events such as hot flashes and excessive sweating, headache, and elevated blood glucose was recorded.

### Statistical analysis:

All statistical analyses were performed using IBM SPSS Statistics 25.0(IBM Corp., Armonk, NY, USA). The confidence interval is 95%. Quantitative data were expressed as mean ± standard deviation (*x̄*+*s*), with independent samples *t*-test for inter-group comparisons and paired samples *t*-test for intragroup comparisons. Enumeration data were expressed as n(%), and intergroup comparisons were conducted using the Chi-square (*χ*^2^) test. A *P*-value <0.05 was considered statistically significant.

## RESULTS

No statistically significant disparities were observed in baseline data between the two groups (*P*>0.05), indicating comparability (Table-I). The overall response rate in the study group was markedly higher than that in the control group (*P*<0.05), as detailed in Table-II.

**Table-I:** Comparison of general data between the two groups [n(%)/(*x̄* + *s*].

Group	Age (years)	Course of disease (months)	BMI (kg/m^2^)	Breast Tanner Stage		
				Stage I	Stage II	Stage III
Control group (n=77)	7.92±0.88	5.79±0.66	14.82±2.29	41 (53.25)	22 (28.57)	14 (18.18)
Study group (n=77)	8.05±0.91	5.91±0.70	15.19±2.46	45 (58.44)	23 (29.87)	9 (11.69)
*χ^2^/t*	0.901	1.094	0.966	1.295		
*P*	0.369	0.275	0.336	0.523		

**Table-II:** Comparison of clinical efficacy between the two groups (n, %).

Group	Markedly Effective	Effective	Ineffective	Overall Response Rate	χ^2^	P
Control group (n=77)	29 (37.66)	34 (44.16)	14 (18.18)	63 (81.82)	4.863	0.027
Study group (n=77)	32 (41.56)	40 (51.95)	5 (6.49)	72 (93.51)		

Following treatment, body height and weight of patients in the study group were significantly higher than those in the control group, while the BA/CA ratio was notably lower in the study group (*P*<0.05), as detailed in Table-III.

**Table-III:** Comparison of growth parameters between the two groups (*x̄* + *s*).

Group	Height (cm)		Weight (kg)		BA/CA	
	Before Treatment	After Treatment	Before Treatment	After Treatment	Before Treatment	After Treatment
Control group (n=77)	1.30±0.15	1.37±0.19^#^	25.31±3.12	31.44±4.20^#^	1.31±0.15	1.15±0.13^#^
Study group (n=77)	1.26±0.14	1.45±0.21^#^	25.09±2.98	34.97±4.58^#^	1.25±0.14	1.01±0.11^#^
*t*	1.711	2.479	0.447	4.985	2.566	7.214
*P*	0.089	0.014	0.655	<0.001	0.011	<0.001

***Note:*** Compared with pre-treatment values in the same group,

# P<0.05.

Following treatment, uterine volume, ovarian volume, and breast size in the study group were significantly smaller than those in the control group (all *P*<0.05), as detailed in Table-IV.

**Table-IV:** Comparison of secondary sexual characteristics between the two groups (*x̄* + *s*).

Group	Uterine Volume (mL)		Breast Size (cm^3^)		Ovarian Volume (mL)	
	Before Treatment	After Treatment	Before Treatment	After Treatment	Before Treatment	After Treatment
Control group (n=77)	4.26±0.55	3.19±0.41^#^	4.47±0.56	3.22±0.45^#^	2.04±0.31	1.70±0.21^#^
Study group (n=77)	4.21±0.52	2.80±0.33^#^	4.41±0.50	2.71±0.36^#^	2.09±0.35	1.38±0.16^#^
*t*	0.580	6.502	0.701	7.766	0.938	10.636
*P*	0.563	<0.001	0.484	<0.001	0.350	<0.001

Following treatment, serum E2, FSH, and LH concentrations in the study group were significantly lower than those in the control group (all *P*<0.05), as detailed in Table-V. No statistically significant difference was observed in the incidence of adverse reactions between the two groups (*P*>0.05), as detailed in Table-VI.

**Table-V:** Comparison of serum sex hormone levels between the two groups (*x̄* + *s*).

Group	E2 (pg/mL)		FSH (mIU/mL)		LH (mIU/mL)	
	Before Treatment	After Treatment	Before Treatment	After Treatment	Before Treatment	After Treatment
Control group (n=77)	19.78±2.64	13.77±1.91^#^	4.74±5.36	3.13±0.37^#^	1.41±0.18	1.10±0.13^#^
Study group (n=77)	19.95±2.71	10.41±1.52^#^	4.59±5.28	2.59±0.31^#^	1.36±0.16	0.92±0.11^#^
*t*	0.394	12.079	0.175	9.817	1.822	9.275
*P*	0.694	<0.001	0.861	<0.001	0.070	<0.001

**Table-VI:** Comparison of adverse reactions between the two groups (n, %).

Group	Hot Flashes and Excessive Sweating	Headache	Elevated Blood Glucose	Overall Incidence Rate	χ^2^	P
Control group (n=77)	2 (2.60)	2 (2.60)	0 (0.00)	4 (5.19)	0.428	0.513
Study group (n=77)	3 (3.90)	2 (3.90)	1 (1.30)	6 (7.79)		

## DISCUSSION

In the present study, the overall response rate in the study group was significantly higher than that in the control group following combined treatment with leuprorelin acetate and rhGH, indicating that this combination regimen exerts a more prominent therapeutic effect in girls with ICPP.^9^ This effect is closely linked to enhanced IGF synthesis; IGF promotes mitosis of cortical bone cells and osteocytes via paracrine/autocrine mechanisms, maintaining a dynamic equilibrium of “bone resorption-bone formation”.^10^ When combined with GnRHa, rhGH compensates for the reduced growth rate induced by GnRHa and improves predicted adult height (PAH) by slowing bone age progression. Prior clinical investigations have demonstrated that the annualized height growth rate of girls in the combined treatment group is approximately 2.5 cm higher than that in the control (GnRHa monotherapy) group, with a significantly lower rate of bone age advancement.^11^ Height development in girls with ICPP follows a “fast-early, slow-late” trajectory, with the core issue being reduced final adult height due to premature epiphyseal closure. In the early stage of the disease, premature HPGA activation increases growth hormone (GH) secretion; coupled with sex hormone-induced bone growth, the height growth rate of girls temporarily accelerates, leading to a false impression of “height advancement”.^12^ The full pathogenic mechanism underlying ICPP remains incompletely elucidated, but current evidence confirms that premature activation of the HPGA is its core pathological basis. Potential contributing factors include genetic polymorphisms, environmental endocrine disruptors, and abnormal neural signal transduction.^13^,^14^ The therapeutic mechanism of GnRHa in treating girls with ICPP involves mimicking sustained GnRH stimulation, which induces pituitary GnRH receptor desensitization, reduces sensitivity to physiological GnRH pulses, and maintains FSH and LH secretion below the pubertal onset threshold. Deprived of sufficient gonadotropin signals, the ovary reduces estrogen secretion, thereby effectively delaying the progression of sexual development. As a sustained-release formulation, GnRHa achieves continuous and stable drug release in the bloodstream via controlled-release technology. Stable plasma concentrations potently inhibit ovarian responsiveness to gonadotropins, exerting a highly effective suppressive effect on the pituitary-gonadal axis, which aligns with the design objectives of long-acting and stable sustained-release agents.^15^ rhGH interacts with growth hormone receptors expressed on the surface of growth plate chondrocytes, activating downstream signaling pathways (e.g., insulin-like growth factor-1 (IGF-1)-mediated proliferation signals) to promote chondrocyte division and proliferation. This process not only accelerates longitudinal growth of long bones but also extends the time window for epiphyseal closure by maintaining normal metabolic activity of the growth plate.^16^,^17^ From the perspective of the hypothalamic-pituitary-growth axis molecular regulatory network, rhGH positively activates osteoclast activity to accelerate bone resorption while directly stimulating the proliferation and differentiation of growth plate chondrocytes during the growth phase.

In our study, we found that post-treatment improvements in body height, weight, BA/CA ratio, uterine volume, ovarian volume, and breast size were all superior in the study group compared to the control group, indicating that the combined regimen of leuprorelin acetate and rhGH can effectively optimize growth parameters and the progression of secondary sexual characteristics in girls with ICPP. In the diagnostic workup of ICPP, alterations in pituitary-derived LH secretion play a pivotal role. As a core regulatory signal for HPGA activation, elevated basal LH concentrations-particularly when exceeding the normal threshold for age-matched girls-directly reflect premature gonadotropin axis initiation. It is an indispensable biological indicator for confirming ICPP, with significant value in pediatric endocrine diagnosis due to its high sensitivity and specificity.^18^ In girls with ICPP, premature HPGA activation often leads to mild elevation of basal FSH levels, which is closely associated with the pathological process of accelerated follicular development.^19^ E2, the primary estrogen, is secreted by ovarian granulosa cells; changes in its levels directly reflect the activation status of ovarian function. The mild elevation of basal E2 levels in girls with ICPP stems from premature ovarian development, serving as an objective marker of early ovarian initiation that corroborates clinical manifestations such as precocious secondary sexual characteristics.^20^ In this study, serum E2, FSH, and LH concentrations were significantly lower in the study group than in the control group, indicating that combined treatment with leuprorelin acetate and rhGH effectively regulates sex hormone levels in girls with ICPP.

As a key hormone secreted by the pituitary gland, FSH provides supportive evidence for the auxiliary diagnosis of ICPP. Although its specificity is relatively low, its core physiological function is to promote the development and maturation of ovarian follicles. Girls with ICPP are often accompanied by weight abnormalities, mainly overweight or obesity: premature HPGA activation alters lipid metabolism via sex hormones (especially estrogen), facilitating fat deposition in the abdomen, buttocks, and other anatomical sites. Some children also have lifestyle-related issues such as excessive energy intake and insufficient physical activity, which form a “bidirectional interaction” with precocious puberty. Bone age is a core indicator of skeletal maturity; a key feature of girls with ICPP is that bone age(BA) is significantly greater than chronological age (CA). The pace of BA advancement directly influences the extent of adult height compromise-if the annual BA progression rate far exceeds that of CA, the epiphyses close rapidly, drastically shortening the growth period. Additionally, the uterus and ovaries of girls with ICPP enter pubertal development prematurely, with increased volume accompanied by endometrial thickening (some children may experience “pseudo-menstruation” 1-2 years after breast development). Follicular development is a direct manifestation of HPGA activation. Breast development is the most intuitive clinical sign in girls with ICPP, typically preceding pubic and axillary hair growth. While some children exhibit slow breast development, others show rapid progression, with the development rate positively correlated with BA advancement and hormone levels.^21^

### Limitations:

However, a small number of sample is the limitation of the study. In view of this, more samples should be included in future studies to further validate the findings of this study.

## CONCLUSIONS

The combined regimen of leuprorelin acetate and rhGH yields substantial therapeutic benefits in girls with ICPP. It may effectively optimize growth parameters and the progression of secondary sexual characteristics, exert favorable regulation of sex hormone levels, and demonstrates favorable clinical safety profiles.

### Authors’ Contributions:

**JX:** Carried out the studies, participated in collecting data, drafted the manuscript, are responsible and accountable for the accuracy and integrity of the work.

**QZ:** Literature search, Performed the statistical analysis and participated in its design.

**YX:** Participated in acquisition, analysis, or interpretation of data and draft the manuscript.

All authors have read and approved the final manuscript.
